# Cellulose Nanofibril-Based Triboelectric Nanogenerators Enhanced by Isoreticular Metal-Organic Frameworks for Long-Term Motion Monitoring

**DOI:** 10.3390/s25103232

**Published:** 2025-05-21

**Authors:** Mingli Shang, Yan Zong, Xiujun Zhang

**Affiliations:** 1 College of Bioresources Chemical and Materials Engineering, Shaanxi University of Science and Technology, Xi’an 710021, China; 220112114@sust.edu.cn; 2 Modern Aviation College, Guangzhou Institute of Science and Technology, Huizhou 516122, China

**Keywords:** CNF, IRMOF, TENG, charge induction, long-term motion monitoring

## Abstract

Cellulose nanofibril (CNF) is a sort of novel nanomaterial directly extracted from plant resources, inheriting the advantages of cellulose as a cheap, green and renewable material for the development of new-generation eco-friendly electronics. In recent years, CNF-based triboelectric nanogenerator (TENG) has attracted increasing research interests, as the unique chemical, morphological, and electrical properties of CNF render the device with considerable flexibility, mechanical strength, and triboelectric output. In this study, we explore the use of isoreticular metal-organic frameworks (IRMOF) as functional filler to improve the performance of CNF based TENGs. Two types of IRMOFs that own the same network topology, namely IRMOF-1 and its aminated version IRMOF-3, are embedded with CNF to fabricated TENGs; their contribution to triboelectric output enhancement, including the roughness effect induced by large particles as well as the charge induction effect arisen from -NH_2_ groups, are discussed. The performance-enhanced CNF-based TENG with 0.6 wt.% of IRMOF-3 is utilized to harvest mechanical energy from human activities and charge commercial capacitors, from which the electrical energy is sufficient to light up light-emitting diodes (LEDs) and drive low-power electronic devices. In addition, a locomotor analysis system is established by assembling the above TENGs and capacitors into a 3 × 3 sensing array, which allowed signal extraction from each sensing unit to display a motion distribution map. These results demonstrate the great potential of CNF/IRMOF-based TENGs for development of self-powered sensing devices for long-term motion monitoring.

## 1. Introduction

Triboelectric nanogenerator (TENG) is emerging as a potential renewable energy source to replace conventional fossil fuel for electricity generation. Its unique mechano-electrical transduction is arisen from contact electrification and electrostatic induction, which is capable to convert ambient energy to electrical power [1,2]. As such, TENG also holds promise for the self-powered sensing technique, because it can harvest energy generated by the movement or vibration from an object, thus eliminating the need for external power to drive the sensor. These features endow TENG with significant potential for innovative applications, such as wave motion monitoring, cathodic protection and pest prevention [3,4], etc.

In recent years, cellulose-based TENGs have shown numerous advantages for practical sensing applications, including human motion detection [5,6], healthcare monitoring [7,8,9,10], and human-machine interaction [11,12], etc. Benefiting from the lightweight and flexibility of polymeric materials, cellulose-based TENGs are extremely suitable for the development of wearable sensing systems with more portable and adaptable designs. As the most abundant and widely distributed green, cost-effective, and renewable material, cellulose has paved a new pathway towards the next-generation eco-friendly TENG with excellent biodegradability and biocompatibility, overcoming the environment and biosafety issues caused by conventional TENGs fabricated by unsustainable synthetic polymers [13].

Structurally, the CNFs prepared via the TEMPO-mediated oxidation method contain –OH and –COOH functional groups [14], of which the lone pair of electrons in oxygen render cellulose good electron-donating ability and triboelectric property (i.e., to lose electrons and become positively charged thereafter) [15]. Since the performance of TENG is directly influenced by the triboelectric layers, a lot of effort has been made to improve the triboelectric properties of cellulosic materials, including chemical modification [16,17,18], dielectric modulation [19], and engineering microstructure on surface [20,21]. These methods aim to adjust the polarity of cellulose to increase surface charge density, or to enlarge contact area through surface morphology control, thereby achieving enhanced electrical output.

Metal-organic frameworks (MOFs) are a class of hybrid materials formed by combining metal ions (or clusters) with multifunctional organic ligands through coordination bonds [22]. Featured for their porous and large specific area, unsaturated metal sites and customizable framework and functional groups, MOFs can be used alone as triboelectric materials or incorporated with polymers to obtain triboelectric composites, exhibiting great potential as active materials to fabricate high-performance TENGs [23,24,25]. Compared with other functional fillers to enhance the performance of TENGs, it avoids the use of toxic organic solvent or corrosive liquid. Although previous works indicated the electrical performance of cellulosic TENG could be improved by introducing functionalized MOFs [26]. the key factors (i.e., roughness or charge-induction effect) that determine the triboelectric property has yet to be elucidated. Besides, the sensory function of TENG-based sensor is limited to detect real-time human motion, that is, the periodic contact-separation of triboelectric layers can only transduce instantaneous mechanical stimuli into on-and-off electrical signals. How to use TENGs for long-term exercise monitoring still remains as an open question.

In this work, we used two types of isoreticular MOFs (IRMOFs), namely IRMOF-1 and IRMOF-3, as active fillers to verify the influence of surface roughness and introduced functional groups on the performance of cellulosic TENG. IRMOF-1 (also known as MOF-5) has [Zn_4_O]^6+^ groups linked to an octahedral array of [O_2_C-C_6_H_4_-CO_2_]_2_ (H_2_BDC) groups, and IRMOF-3 is the resultant of -NH_2_ substitution reaction in the benzene ring of H_2_BDC groups [27,28]. The above two fillers were embedded with cellulose nanofibrils (CNFs), respectively, to fabricated CNF/IRMOF tribo-positive layers. Their surface roughness and the amount of introduce -NH_2_ groups were controlled by the particle size and different weight ratio percentages of added fillers. IRMOFs hold some specific advantages when comparing with other enhancement materials in TENGs. First, they can be prepared in aqueous solution, thus avoiding the environmental risk caused by the use of toxic organic solvents or corrosive liquids for the preparation and exfoliation of nano-materials (e.g., PVDF and MXene) [29]. Moreover, IRMOFs allow in situ synthesis process in the suspension of CNFs and therefore solve the uneven distribution or aggregation problems faced by nano-fillers (e.g., carbon nanotubes) [30]. In addition, the polarized -NH_2_ in IRMOF-3 plays as electro-releasing group, of which the unique charge induction property is expected to further enhance the performance of CNF-based TENG. As such, various experiments were performed to characterize the structure and morphology of obtained triboelectric composites, and their triboelectric properties were tested as well. The optimized CNF/IRMOF TENG was used to light up LEDs as a real-time movement indicator, and nine TENGs were assembled into a sensing array for long-term motion monitoring. This work demonstrated that IRMOFs, due to controllable morphology and variety of functional groups, can be used to fabricate reliable cellulose TENGs with great potential for human motion and intelligent sports applications.

## 2. Experimental Section

### 2.1. Materials

Sodium bromide, sodium chloride, zinc chloride, and sodium hypochlorite were purchased from Tianjin Tianli Chemical Reagent Co., Ltd. (Tianjin, China). Sodium hydroxide was purchased from Tianjin Damo Chemical Reagent Co., Ltd. (Tianjin, China). Hydrochloric acid was purchased from Xi’an Sanpu Fine Chemicals Factory (Xi’an, China). Methanol and triethylamine were purchased from Tianjin Fuyu Fine Chemical Co., Ltd. (Tianjin, China). Anhydrous ethanol was purchased from Tianjin Oubokai Chemical Co., Ltd. (Tianjin, China). Potassium bromide was purchased from Tianjin Kemiou Chemical Reagent Co., Ltd. (Tianjin, China). Zinc nitrate hexahydrate was purchased from Tianjin Fengchuan Chemical Reagent Technology Co., Ltd. (Tianjin, China). 2,2,6,6-tetramethylpiperidine-1-oxyl radical (TEMPO), terephthalic acid, and 2-aminoterephthalic acid were purchased from Shanghai Macklin Biochemical Technology Co., Ltd. (Shanghai, China). All chemicals used in this study were of analytical grade and required no further purification.

### 2.2. Preparation of IRMOF-1 and IRMOF-3 Crystals

Terephthalic acid and 2-amino terephthalic acid were employed as aromatic acid ligands, with zinc nitrate hexahydrate as the zinc source, to synthesize two distinct IRMOFs via a traditional solvothermal method. In detail, to synthesize IRMOF-1, 1.67 g of zinc nitrate hexahydrate and 0.35 g of terephthalic acid were dissolved in 40 mL of N, N-dimethylformamide (DMF) and sonicated until fully dissolved; the mixture was then reacted at 90 °C for 4 h and cooled down to room temperature, and then the solution was centrifuged at 10,000 rpm for 8 min, followed by washing the resulting precipitate with DMF three times. The obtained IRMOF-1 was dried at 60 °C for 24 h to yield white crystals. To synthesize IRMOF-3, 1.20 g of zinc nitrate hexahydrate and 0.33 g of 2-amino terephthalic acid were dissolved in 40 mL of DMF. Triethylamine (1.6 g) was added dropwise, stirring the mixture at room temperature for 12 h. The precipitate was washed three times with DMF and dried at 60 °C for 24 h, yielding chartreuse crystals, identified as IRMOF-3.

### 2.3. Preparation of Cellulose Nanofibrils (CNFs)

CNFs were prepared following the procedure reported elsewhere [31]. Briefly, 1 g of bleached hardwood pulp was immersed in deionized water and stirred vigorously for 6 h to obtain a well-dispersed kraft pulp. The pulp was then filtered and transferred into a 100 mL solution containing 0.15 mol of 2,2,6,6-tetramethylpiperidine-N-oxide (TEMPO) and 1.2 mol of sodium bromide (NaBr). A sodium hypochlorite (NaClO) solution, at a concentration of 6–14 wt.%, was added dropwise to initiate the oxidation reaction. Sodium hydroxide (NaOH) was added to maintain the mixture’s pH around 10, allowing the reaction to proceed at room temperature for 6 h. Once the pH stabilized, 10 mL of ethanol was added to terminate the reaction, and the pH was adjusted to 7 using 0.5 mol of hydrochloric acid (HCl). The oxidized CNF suspension was washed three times with deionized water, followed by high-speed homogenization and cell disruption, yielding a 1 wt.% CNF aqueous suspension which was stored at 4 °C for further use.

### 2.4. Preparation of CNF/IRMOF-1 and CNF/IRMOF-3 Composite Films

CNF/IRMOF-1 and CNF/IRMOF-3 dispersions were prepared at first by mixing 1 wt.% CNF suspension with different contents of IRMOF-1 and IRMOF-3 crystals, respectively, and then stirred at room temperature at 200 rpm for 12 h to obtain viscously uniform dispersions. After this, the dispersions were poured into hydrophobic molds and evaporated in a vacuum oven at 60 °C to produce CNF/IRMOF-1 and CNF/IRMOF-3 composite films with 0, 0.2, 0.4, 0.6, and 0.8 wt.% of filler contents.

### 2.5. Characterization and Electrical Measurement

A scanning electron microscope (SEM, Hitachi S-4800, Ibaraki, Japan) was operated at an accelerating voltage of 5.0 kV to observe nanoparticle structures and the surface morphology of the films. Energy-dispersive X-ray spectroscopy (EDS) was used to analyze elemental distribution and content in selected areas. X-ray diffraction (XRD, BRUKER, Karlsruhe, Germany) and Fourier-transform infrared spectroscopy (FT-IR, BRUKER, Karlsruhe, Germany) characterized the crystalline and chemical structures of IRMOF, CNF films, and CNF/IRMOF composite films, with an XRD scanning angle of 5° to 50° and an FT-IR spectral range of 4000 to 400 cm^−1^. The tensile strength and elongation at the break of CNF/IRMOF composites were measured using a servo-system tensile testing machine (TS-2000, Highspeed Technology, Taichung, China). 3-D surface morphology maps of CNF and CNF/IRMOF composite films were obtained using a digital microscope (SDOFM, KH 8700, Tokyo, Japan). Additionally, an electrometer (Keithley-6514, Beavertonm, OR, USA) was used to measure the triboelectric output (open-circuit voltage and short-circuit current) of TENGs, and pressure-sensing and energy-harvesting applications were tested with the linear-motor acquisition system (NTI AG HS01). All output signals were recorded by custom-made software.

## 3. Results and Discussion

### 3.1. Structure and Morphology Characterizations of CNF/IRMOF Composite Films

**Figure 1:**
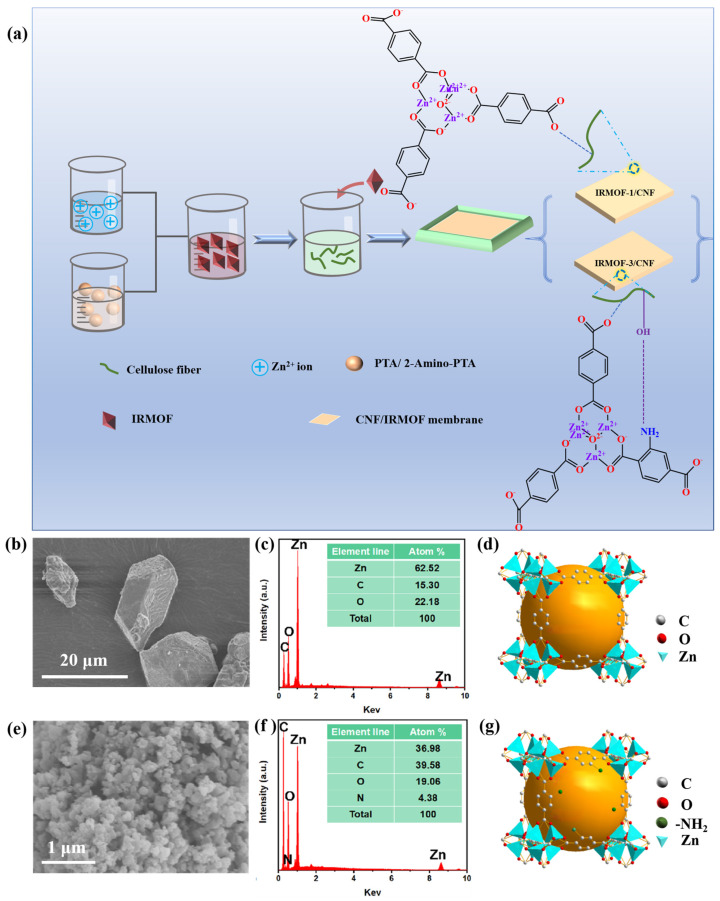
Preparation of IRMOFs and CNF/IRMOFs Composite Films. (**a**) Schematic illustration of the preparation process of IRMOFs and CNF/IRMOF composite films; (**b**) SEM image and (**c**) EDS spectrum of IRMOF-1 crystals; (**d**) schematic view of IRMOF-1 network; (**e**) SEM image of and (**f**) EDS spectrum of IRMOF-3 crystals; (**g**) schematic view of IRMOF-3 network.

Figure 1a illustrates the preparation process of CNF/IRMOF Composite Films. First, IRMOF-1 and IRMOF-3 were prepared by connecting [Zn_4_O]^6+^ to TPA or 2-aminoterephthalic acid (2-Amino-TPA), respectively. Then, the obtained IRMOF fillers were blended with CNFs to fabricate CNF/IRMOF-1 and CNF/IRMOF-3 composites with different filler contents (0–0.8 wt.%). The composite films were referred to as CI-1 0 wt.%, CI-1 0.2 wt.%, CI-1 0.4 wt.%, CI-1 0.6 wt.%, and CI-1 0.8 wt.% for the CNF/IRMOF-1 series, and similarly, CI-3 0 wt.%, CI-3 0.2 wt.%, CI-3 0.4 wt.%, CI-3 0.6 wt.%, and CI-3 0.8 wt.% were used to label the CNF/IRMOF-3 series.

To investigate the effect of roughness and chemical modification of IRMOF on triboelectric output, we synthesized IRMOF-1 and IRMOF-3 with different particle sizes by controlling the reaction conditions. In detail, IRMOF-1 crystals were selectively prepared into larger particles of which the diameter is from 5–10 μm (Figure 1b), and by doing so a small amount of filler addition could cause significant morphology change on the CNF/IRMOF-1 surface. The relative percentages of Zn, C, and O were 62.52%, 15.30%, and 22.18%, respectively (Figure 1c) in IRMOF-1. Comparably, the aminated IRMOF crystals, IRMOF-3, was employed as functional fillers to study the charge induction effect arisen from -NH_2_ groups. To reduce the influence of roughness, the particle size of IRMOF-3 was controlled within 100 nm (Figure 1e), and the relative distribution of Zn, C, O, and N elements were found as 36.98%, 39.58%, 19.06%, and 4.38%, respectively (Figure 1f). The synthesized IRMOF-1 and IRMOF-3 were constructed from octahedral Zn-O-C clusters and varied organic ligands (i.e., TPA and 2-Amino-TPA), sharing the similar pcu topology as shown in Figure 1d,g). Figure S1a,b show the appearance of both IRMOF-1 and IRMOF-3, and it was clear the IRMOF-1 appeared as white powder while its aminated form, IRMOF-3, turned into chartreuse color. The successful preparation of IRMOF-1 and IRMOF-3 was further confirmed by XRD and FT-IR. As can be seen from FT-IR spectra (Figure S2a), both IRMOFs exhibit C-H out-of-plane bending vibrations in 833–661 cm^−1^ and in-plane bending vibrations in 1250–1000 cm^−1^. Specifically, symmetric solid and asymmetric stretching vibrations of the ligand 2-Amino-TPA appear at 1610–1550 cm^−1^ and 1420–1375 cm^−1^, respectively (red curve, IRMOF-3). The double-peak band around 3400 cm^−1^ is associated with amine group stretching vibrations, while the absorption at 1252 cm^−1^ corresponds to C-N stretching, confirming the existence of -NH₂ groups in IRMOF-3. Figure S2b displays the X-ray diffraction (XRD) patterns of the two types of IRMOF crystals, in which the five diffraction peaks at 2θ = 9.2°, 11.5°, 13.6°, 16.9°, and 20.8° correspond to the (200), (220), (400), (420), and (531) crystal lattices of IRMOF-1; for IRMOF-3 the distinct diffraction peaks at 2θ = 6.8°, 9.6°, 13.7°, and 15.4° correspond to the (200), (220), (400), and (420) lattices, which have a good agreement with previously reported results [32].

CNF produced by the 2,2,6,6 tetramethyl piperidinyloxy (TEMPO) oxidation method was employed as the main component of the tribo-positive layer of TENG. Figure S3a is the Atomic Force Microscope (AFM) image representing the morphology of two parallel CNFs, and Figure S3b shows the cross-sectional profile of a single CNF obtained from the location labeled in green color. From the AFM scanning results, the diameter of the CNF was determined as 7.5 nm and the length of the CNF can reach up to ~1 μm. The abundant -COO^-^ surface groups can provide strong static repulsions between CNFs, thus resulting with very stable CNF suspension in water. Meanwhile, the interplays between CNF and IRMOFs, such as the hydrogen bonds established between carboxyl groups on CNF and TPA of IRMOF-1, respectively, as well as the hydrogen bonds between -NH_2_ on IRMOF-3 and -COO^-^ on CNFs, could enhance matrix-filler interactions. Therefore, the blended CNF/IRMOF-1 and CNF/IRMOF-3 with different filler contents can be well-dispersed in aqueous conditions, as shown in Figure S4a–j.

The aforementioned suspensions were then evaporated in a vacuum oven to remove bubbles and form CNF/IRMOF-1 and CNF/IRMOF-3 composite films. However, it is worth noting that the CNF/IRMOF-1 films, no matter the amounts of added fillers, had wrinkled surfaces and outward curl on the outer edges, as shown in Figure S5a. This phenomenon was probably caused by the mechanical mismatch between the large IRMOF-1 fillers and the rigid CNFs, that is, during the evaporation process, the CNFs were not possible to maintain their original arrangement due to the obstruction of large fillers. As a result, the increased internal stress caused rearrangement of CNFs, and the forming films no longer had smooth surfaces. To solve this, we used ZnCl_2_ to optimize the film-formation process, as Zn^2+^ could coordinate with -COO^-^ groups on CNF thus stabilizing the network structure of CNF/IRMOF. After the addition of ZnCl_2_ solution, Zn^2+^ quickly diffused in CNF/IRMOF mixtures, and a clear gelation process was observed where the large fillers of IRMOF-1 were firmly incorporated into the CNF matrix. Thereafter, the CNF/IRMOF-1 prepared by the optimized method with a flattened surface was obtained, as shown in Figure S5b.

**Figure 2:**
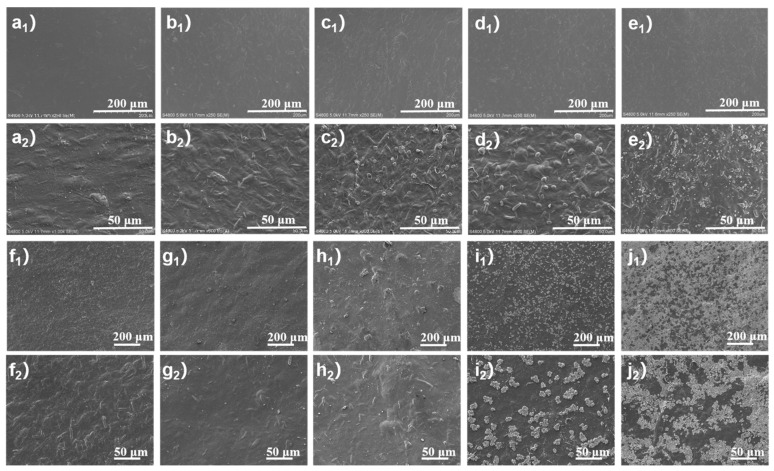
Surface morphology of CNF/IRMOF-1 and CNF/IRMOF-3 composite films with different filler contents. (**a**–**e**) SEM images of CNF/IRMOF-1 surfaces with (**a**) 0, (**b**) 0.2, (**c**) 0.4, (**d**) 0.6, and (**e**) 0.8 wt.% of IRMOF-1, respectively; (**a_1_**–**e_1_**) are at 250× magnification and (**a_2_**–**e_2_**) are at 600× magnification. SEM images of CNF/IRMOF-3 surfaces with (**f**) 0, (**g**) 0.2, (**h**) 0.4, (**i**) 0.6, and (**j**) 0.8 wt.% of IRMOF-3, respectively; (**f_1_**–**j_1_**) are at 250× magnification and (**f_2_**–**j_2_**) are at 600× magnification.

The surface roughness of triboelectric material is accounted as one of the key factors influencing the performance of TENG, because higher roughness provides more contact area for triboelectric charge generation, leading to enhanced electrical output [33]. To study the roughness change induced by added fillers, we use Scanning Electron Microscope (SEM) to observe the morphology of CNF/IRMOF-1 and CNF/IRMOF-3 composite films with different filler contents. In detail, Figure 2(a_1_–e_1_) is a SEM image showing the overall appearance of CI-1 0 wt.%, CI-1 0.2 wt.%, CI-1 0.4 wt.%, CI-1 0.6 wt.%, and CI-1 0.8 wt.% films, and the corresponding magnified morphologies could be found in Figure 2(a_2_–e_2_). By comparing Figure 2(a_2_,b_2_), it was noticed that the addition of 0.2 wt.% of IRMOF-1 did not cause obvious change of surface morphology, indicating the fillers were mainly incorporated in the CNF matrix. However, when the filler content exceeded 0.4 wt.%, IRMOF-1 particles were observed on the surfaces and their quantity gradually increased according to the rise of filler concentration. The overall and zoom-in morphologies of CI-3 0 wt.%, CI-3 0.2 wt.%, CI-3 0.4 wt.%, CI-3 0.6 wt.%, and CI-3 0.8 wt.% films can be found in Figure 2(f_1_–j_1_) and Figure 2(f_2_–j_2_), respectively. Since the particle size of IRMOF-3 is much smaller than IRMOF-1, the surface morphology was not significantly change until the filler content reached up to 0.4 wt.%. In addition, IRMOF-3 aggregated as surface colonies at higher concentration (i.e., 0.6 and 0.8 wt.%), rather than the single particles of IRMOF-1, which were observed at the surface of the CI-1 0.6 wt.% and CI-1 0.8 wt.% films.

**Figure 3:**
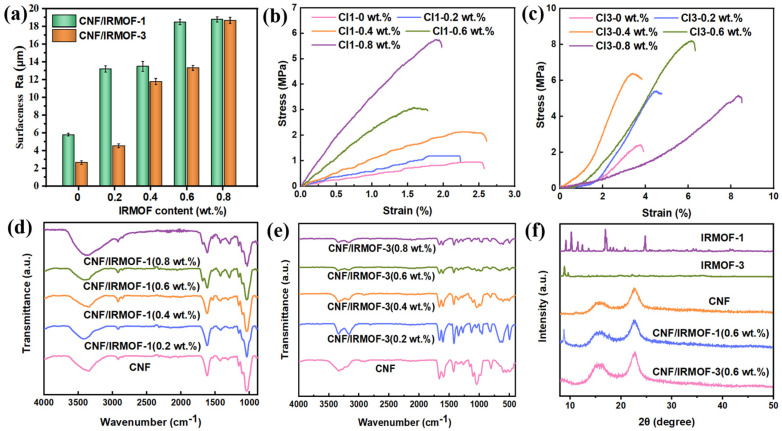
(**a**) Surface roughness (Ra) change of CNF/IRMOF-1 and CNF/IRMOF-3 composite films according to different IRMOF contents; stress-strain curves of (**b**) CNF/IRMOF-1 and (**c**) CNF/IRMOF-3 composite films with different filler contents; FT-IR spectra of (**d**) CNF/IRMOF-1 and (**e**) IRMOF-3 composite films with different filler contents; (**f**) XRD patterns of IRMOF-1, IRMOF-3, CNF, CNF/IRMOF-1, and IRMOF-3 composite films with 0.6 wt.% filler content.

We also used a digital microscope to build up 3-D morphology maps for the above CNF/IRMOF-1 and CNF/IRMOF-3 composite films (Figure S6) through a layer-by-layer scanning technique, and the roughness data was summarized in Figure 3a. Due to the larger particle size of IRMOF-1, the surface roughness of CNF/IRMOF-1 films grew up more rapidly than CNF/IRMOF-3 films when the filler content increased from 0 to 0.6 wt.%. Finally, when 0.8 wt.% of IRMOFs were added, both CI-1 0.8 wt.% and CI-3 0.8 wt.% films reached the maximum roughness of ~19 μm. Figure 3b,c show the tensile testing results of CNF/IRMOF-1 and CNF/IRMOF-3 composite films with varying IRMOFs contents. Briefly, the tensile strength of CNF/IRMOF-1 films was enhanced according to increasing filler contents, indicating the hydrogen bonds could anchor the fillers to CNFs and stabilize the film structure. Although the highest tensile strength of 5.7 MPa was achieved at filler content of 0.8 wt.%, it should not be ignored that the fracture strain was reduced to 2.0%, again proving the mechanical mismatch between the large particles of IRMOF-1 and the CNF matrix as discussed above. Comparably, IRMOF-3 exhibited a more obvious anchoring effect than IRMOF-1 as the highest tensile strength of 8.1 MPa was determined for the CI-3 0.6 wt.% sample; meanwhile, its fracture strain was increased to 6.0%. We also noticed that the tensile strength of the CI-3 0.8 wt.% film was decreased to 4.4 MPa, which was probably attributed to the aggregated IRMOF-3 seen in Figure 2(j_1_,j_2_). Since IRMOF-3 particles trended to get together at the surface of the CI-3 0.8 wt.% film, it is reasonable to presume the similar aggregation also occurred in the interior of the film. On one hand, the aggregation was easily to decompose under high stress; on the other, these loosely attached particles could serve as a sacrificial structure for energy dissipation, and therefore caused an increased fracture strain up to 8.4%.

From the tensile testing results, it was clear that IRMOFs played a role as an anchor to reinforce the structure of the CNF network. However, there still laid the possibility that the mechanical mismatch could weaken the interaction between IRMOF-1 and CNF matrix. To verify this, FT-IR was employed to reveal the interplays between IRMOF-1/IRMOF-3 and CNFs. As displayed in Figure 3d,e, the absorption band at 2923 cm^−1^ associated with symmetric stretching of C-H was found in pure CNF and all CNF/IRMOF films, suggesting IRMOFs did not affect the C-H vibration on cellulose. However, the strong interaction between IRMOF-3 and CNFs restrained the vibration of -OH on cellulose, as the absorption band at 3335 cm^−1^ (-OH stretching) was dramatically reduced with the added fillers. In contrast, the absorption band of -OH was not changed in all CNF/MOF-1 composite films. It was also observed that the absorption band at 1020 cm^−1^, which is attributed to the stretching vibration of C-O-C glycosidic bond, gradually reduced upon the increasing IRMOF-3 content while the addition of IRMOF-1 did not show obvious influence on this absorption band. The FT-IR spectra has evidenced that the stronger interaction between IRMOF-3 and CNFs limited the movement of cellulose. On the contrary, the incorporation of larger IRMOF-1 particles left enough space in the CNF matrix, thus allowing the movement polymer chains. The XRD results also suggested the incorporated IRMOFs did not affect the original crystalline structure of CNF, as shown in Figure 3f. The feature peaks corresponding for IRMOF-1 and IRMOF-3 were all observed in the representative composite films, CI-1 0.6 wt.% and CI-3 0.6 wt.%, respectively, implying the coexistence of IRMOFs and CNFs in both two composites. Moreover, the peaks of (101), (10$\tilde{1}$), (002), and (004) lattices assigned to cellulose crystal I at 2θ = 14.9, 17.4, 22.7, and 34.6°, respectively [34], all remain unchanged compared to the XRD pattern of pure CNF.

### 3.2. Structure and the Working Mechanism of CNF/IRMOFs TENGs

**Figure 4:**
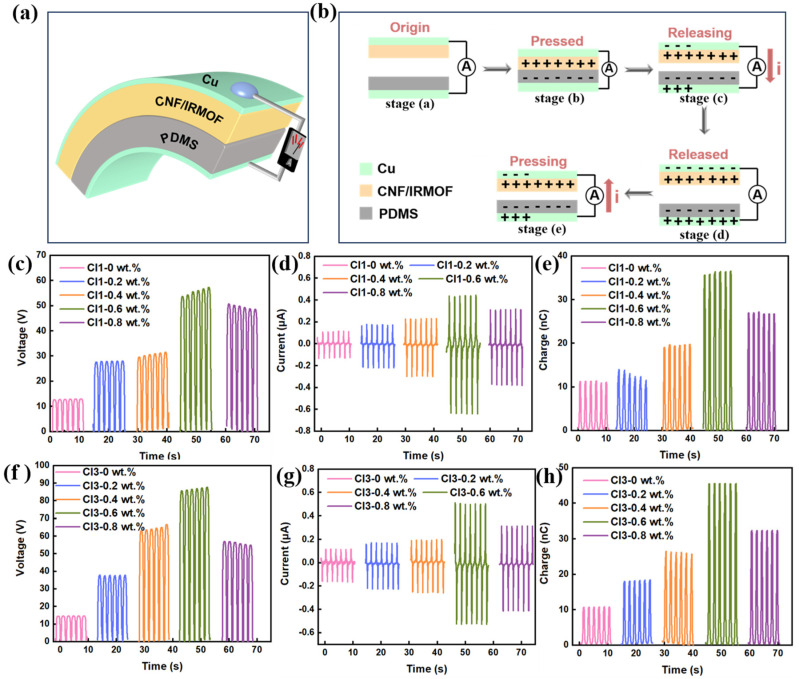
Schematic illustration and electrical performance of CNF/IRMOFs-based TENGs. (**a**) Schematic illustration of CNF/IRMOFs-based TENG construction and (**b**) the working mechanism. (**c**–**e**) Open-circuit voltage, short-circuit current, and transferred charge of CNF/IRMOF-1-based TENGs (CI-1 series) with different IRMOF-1 contents under 20 N pressure, 1 Hz stimulating frequency, and 10 mm of separation distance; (**f**–**h**) Open-circuit voltage, short-circuit current, and transferred charge of CNF/IRMOF-3-based TENGs (CI-3 series) with different IRMOF-3 contents under 20 N pressure, 1 Hz stimulating frequency, and 10 mm of separation distance.

The prepared CNF/IRMOF-1 and CNF/IRMOF-3 composite films with different filler content were employed as tribo-positive layer, which were then combined with Polydimethylsiloxane (PDMS), respectively, to fabricated TENGs (Figure 4a). It should be pointed out that the purpose of this study was to elucidate the roughness and induction effects of IRMOFs on the performance of TENGs rather than pursuing high electrical output. In this case, the plastic materials with strong electron acquisition capability in the electro negativity sequence [35], e.g., PVDF and its co-polymers, were not selected as the tribo-negative material. Instead, an elastomeric material PDMS was chosen because only in this way the tribo-negative layer can tightly contact with the rough surface of CNF/IRMOF films.

All CNF/IRMOF-based TENGs were designed in contact-separation mode, and the working mechanism is displayed in Figure 4a. At the beginning stage (stage (a) in Figure 4a), neither triboelectric layer had charges generated. When the layers came into contact under external force, the CNF/IRMOF layer was likely to lose electrons while the PDMS layer was likely to gain electrons. This electron transfer process resulted in positive charges accumulating on the CNF/IRMOF surface and negative charge accumulating on the PDMS surface (stage (b) in Figure 4b). When the direction of the external force was reversed, the separated upper and bottom layers began to create a potential difference, which drove current that flowed through the external circuit from the surface electrode attached on CNF/IRMOF film to the bottom electrode attached on PDMS (stage (c) in Figure 4b). The equilibrium condition was reached when the separation of CNF/IRMOF and PDMS achieved the maximum distance (stage (d) in Figure 4b). After this, the direction of external force changed again, and subsequently, the two triboelectric layers got closer to each other, causing a decrease of potential difference and generating a backward current flowing from the bottom electrode back to the surface electrode through the external circuit (stage (d) in Figure 4b). Thus, during periodic contact and separation, the current alternates direction, enabling the external circuit to generate a continuous alternating signal that converts mechanical energy into electrical energy.

### 3.3. Roughness and Charge Induction Effects on TENG Performance

To elucidate the roughness and charge induction effects of IRMOFs on TENG performance, the open-circuit voltage, short circuit current, and transferred charge of CNF/IRMOF-1 and CNF/IRMOF-3-based TENGs were recorded. A linear-motor acquisition system was employed to apply a cyclic pressure of 20 N to drive two triboelectric layers in periodic contact-separation at a frequency of 1 Hz. For all TENGs, the maximum distance at separation status was fixed at 10 mm. As shown in Figure 4c–e, the triboelectric output of the CNF/IRMOF-1 based TENG initially increased with rising IRMOF-1 content, reaching the optimum values at 0.6 wt.% of IRMOF-1 with the open-circuit voltage of 54.1 V, the short-circuit current of 0.45 μA, and the transferred charge of 36.4 nC, representing 350%, 260%, and 220% improvements over pure CNF-based TENG, respectively. Similarly, the CNF/IRMOF-3-based TENGs also exhibited an output-enhancement effect with increasing IRMOF-3 content. However, the addition of IRMOF-3 resulted in a more significant improvement in TENG performance. As shown in Figure 4f–h, the peak values of open-circuit voltage of 87.6 V, short-circuit current of 0.51 μA, and transferred charge of 45.4 nC were recorded from the CI-3 0.6 wt.% TENG, corresponding to 504%, 218%, and 316% improvements over the CI-3 0. wt.% TENG containing only pure CNFs. Interestingly, declines in open-circuit voltage, short-circuit current, and transferred charge were observed from both CI-1 0.8 wt.% and CI-3 0.8 wt.% TENGs. This negative impact on TENG performance is probably caused by the large amounts of exposed particles which covered CNFs, thus hindering the sufficient contact between the triboelectric components. It also needs to mention that the CI-1 0 wt.% film, though only containing pure CNFs, was also prepared using the same method for casting other CI-1 films, in which ZnCl_2_ was introduced to prevent curling and wrinkling. From the results shown in Figure 4c–h, it was found there was almost no difference in triboelectric output between the CI-1 0 wt.% and CI-3 0 wt.% TENGs, suggesting the addition of ZnCl_2_ imposed no influence on TENG performance.

From the above results, it can be concluded that IRMOFs can improve TENG performance through two pathways. First, the increase of triboelectric output of CI-1 0 to 0.6 wt.% has a good agreement with the tendency of roughness change, confirming the roughness effect played a critical role for TENG performance enhancement. Second, although the triboelectric output of CI-3 0 to 0.6 wt.% is also proportional to roughness increase, the charge induction effect of -NH_2_ groups is the dominant factor that influenced TENG performance. To be more specific, the roughness of CNF/IRMOF-1 films increases faster than that of the CNF/IRMOF-3 films (Figure 3a), and if there are no other factors affecting the output, CNF/IRMOF-1-based TENGs should have better performance than CNF/IRMOF-3-based TENGs. However, each TENG in the CI-3 series performed much better than their counterparts in the CI-1 series, indicating that in addition to roughness effect, charge-induced effect played a more significant role in the improvement of triboelectric output.

### 3.4. Applications of CNF/IRMOF TENG

**Figure 5:**
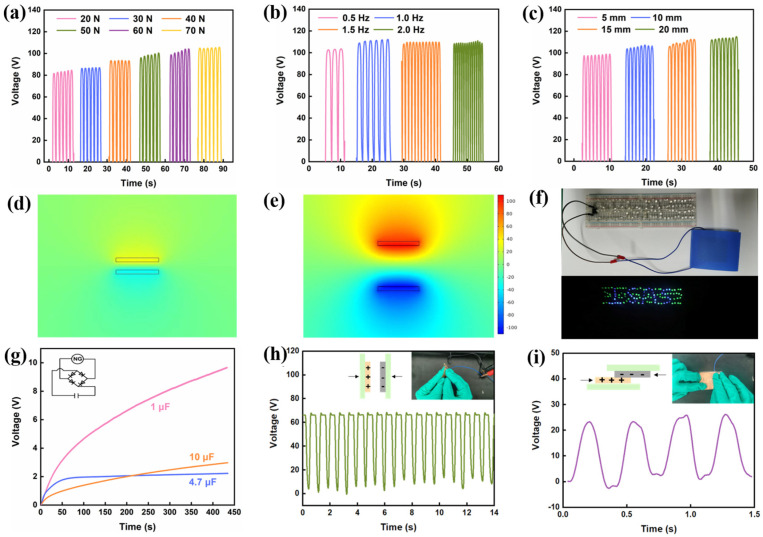
Simulation and optimization of the CI-3 0.6 wt.% TENG. Open-circuit voltage of the CI-3 0.6 wt.% TENG operated under (**a**) different cyclic pressures, (**b**) different stimulating frequencies and (**c**) at different separation distances. (**d**–**e**) COMSOL simulations of the CI-3 0.6 wt.% TENG at contact and separation status. (**f**) Photo of the CI-3 0.6 wt.% TENG lighting up LED bulbs. (**g**) Voltage curves of 1 μF, 4.7 μF, and 10 μF capacitors charged by the CI-3 0.6 wt.% TENG. Application of the CI-3 0.6 wt.% TENG for real-time human motion monitoring in (**h**) contact-separation and (**i**) lateral-sliding modes.

Besides the inherent properties that can influence the triboelectric output, we also investigated the effects of other factors on TENG performance. As shown in Figure 5a, when the applied force increased from 20 N to 70 N, the open-circuit voltage increased from 84.1 V to 104.8 V, and the enhanced output could be attributed to enlarged contact area under high pressure. The pressure higher than 70 N has not been attempted owing to the limitation of the linear-motor acquisition system. In order to find the optimum operation conditions, the maximum pressure of 70 N was applied for all the following tests. Figure 5a presents the influence of contact-separation frequencies on triboelectric outputs, where the relatively low frequency of 0.5 Hz generated the lowest voltage ~100 V. When the stimulating frequency increased above 1 Hz, the output voltage no longer increased and stayed at 110 V (Figure 5b), indicating the upper limit of electron transfer efficiency was achieved at 1 Hz. As aforementioned, the distance between two triboelectric layers determines the potential difference and thereby affects triboelectric output [36]. Accordingly, it was found that the output voltage was increase from 100 to 115 V when the distance at maximum separation status changed from 5 to 20 mm (Figure 5c), and the relationship between output voltage and separation distance could be expressed by the following equation:

(1) $$V_{\mathrm{oc}}=\frac{\sigma x\left(t\right)}{\varepsilon_{0}}$$

where V_oc_ represents the open-circuit voltage, σ is the surface charge density, x(t) is the distance between the positive and negative triboelectric layers, and ε_0_ is the vacuum permittivity. Knowing this, we used multi-physics simulation software (COMSOL, version 6.1) to simulate the electrical properties of CNF/IRMOF TENG at contact and separation conditions, respectively (Figure 5d,e). By setting the maximum separation distance between the two triboelectric layers to 5 mm, the potential distribution around the material was calculated to be approximately ±100 V, which is consistent with the actual testing results.

Due to its unique mechano-electrical transduction property, TENG has the ability to harvest mechanical energy and exhibits great potential for self-powered portable electronics. Figure 5f shows the application of using CNF/IRMOF TENG as a power source. The assembled CI-3 0.6 wt.% TENG could harvest mechanical energy from human motion and easily illuminate over 40 LED bulbs (see Videos S1 and S2). In practical electronic applications, it is necessary to use a bridge rectifier circuit to convert the alternating current generated by the TENG into direct current and then store in a capacitor. We also used the CI-3 0.6 wt.% TENG to charge different commercial capacitors of 1 μF, 4.7 μF, and 10 μF capacities through a commercial bridge rectifier. As shown in Figure 5g, the voltage loaded on the capacitors increased rapidly at initial stage, reaching up to 9.6 V, 2.2 V, and 2.9 V within 430 s, respectively. To further explore the feasibility of using the CI-3 0.6 wt.% TENG for real-time human motion monitoring, we compared the triboelectric voltage output of CI-3 0.6 wt.% TENG generated by contact-separation and lateral-sliding modes, as shown in Figure 5h,i. The results showed that the vertical impact could induce an output voltage of 65 V while the voltage generated from horizonal movement was 24 V, suggesting that energy-harvesting efficiency under contact-separation mode was superior to that under lateral-sliding mode.

**Figure 6:**
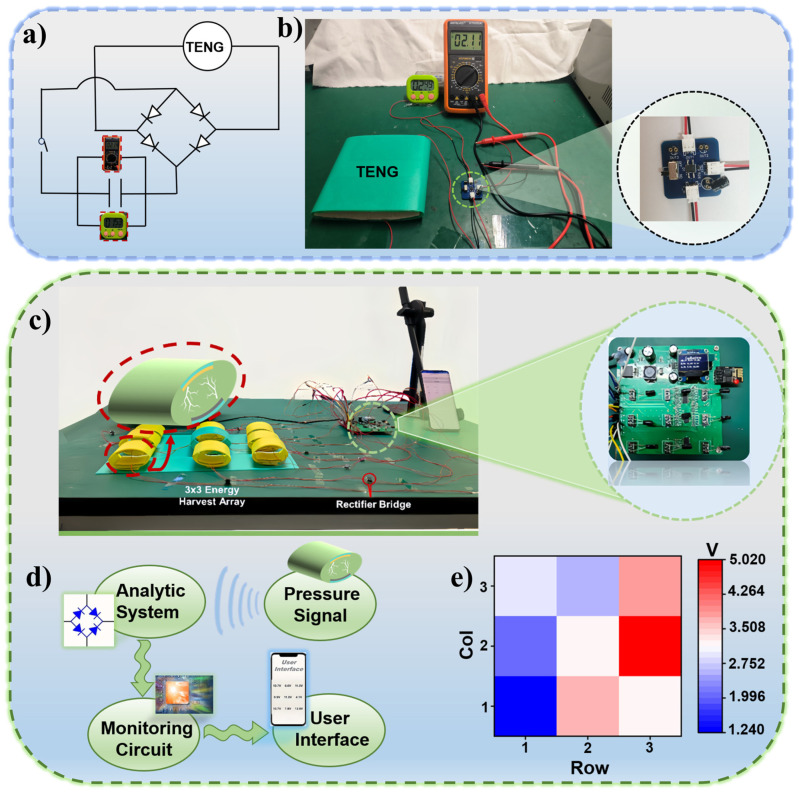
Applications of the CI-3 0.6 wt.% TENG. (**a**) Electrical circuit of the CI-3 0.6 wt.%, a capacitor and a bridge rectifier for powering a timer. (**b**) Photo of the CI-3 0.6 wt.% TENG powering a timer. (**c**) Photo of the analytic system for long-term motion monitoring, which involves three parts: a 3 × 3 energy harvesting array of CI-3 0.6 wt.% TENGs, a monitoring circuit for signal acquisition, and Bluetooth transmission to connect personal electronic devices. (**d**) Schematic diagram of signal acquisition by each sensing unit and the output signal to user interface. (**e**) Visualize motion distribution map showing the hotspots of human activities.

For practical implementation, CI-3 0.6 wt.% TENG was used to drive a low-power electronic device with a 1 μF capacitor and the schematic diagram of circuit was displayed in Figure 6a,b is the photograph of CI-3 0.6 wt.% TENG connected with a timer, showing that the output voltage could drive the device efficiently (see Video S3). We also attempted to expand TENG’s energy harvesting ability to locomotor activity analysis. However, it should be noted that the periodic contact-separation of triboelectric layer only provides real-time on-off signals, which is in conflict with the desire for long-term motion monitoring. To solve this, we designed a 3 × 3 energy harvesting array consisted of nine CI-3 0.6 wt.% TENGs, where the three rows of TENGs were defined as row 1, row 2, and row 3 from left to right, and the three columns of TENGs were column 1, column 2, and column 3 from bottom to top (Figure 5c). Moreover, variations in ambient humidity have minimal impact on the electrical output of the TENG, indicating its excellent output stability under different environmental conditions (Figure S7). The motion monitoring function is realized by connecting each TENG to a capacitor involved in an analytic system. When a sensing unit was touched, the voltage loaded to the corresponding capacitor was recorded and the location of the unit was then confirmed (Figure 5d). The output from the monitoring circuit could be transferred to the user interface, as shown in Figure 5e. The motion-distribution map could be built up based on the voltage analysis results. By referring to the color key, it was determined that the intersection of Col 2 and Row 3 is the most frequently touched location.

## 4. Conclusions

In this study, IRMOF-1 and IRMOF-3 were used as functional fillers to elucidate the roughness and charge-induction effects on the performance of CNF-based TENGs. IRMOF-3 with smaller particle size showed less influence on surface roughness. However, all TENGs containing different weight ratio percentage of IRMOF-3 exhibited superior triboelectric output to their counterparts incorporated with IRMOF-1, indicating the charge-induction effect arisen from -NH_2_ groups dominated the triboelectric improvement. The CNF/IRMOF-3 TENG with optimized filler content of 0.6 wt.% generated an open-circuit voltage of 87.6 V, short-circuit current of 0.51 μA, and transferred charge of 45.4 nC, respectively. It was feasible to harvest mechanical energy from human activities to charge capacitors and the highest voltage, which could be further enhanced to 115 V, is capable to illuminate LED bulbs or drive low-power electronic devices. In addition, an analytic system involving a 3 × 3 array of CI-3 0.6 wt.% TENGs and capacitors was established for locomotor activity analysis, allowing voltage signal extraction from each sensing unit to display the motion distribution. The sensing array can effectively convert instantaneous signal to cumulative signals, thus making it extremely suitable for long-term sensing applications. This work demonstrated that by taking the merits of IRMOF fillers the CNF/IRMOF-based TENG obtained dramatically improved triboelectric output, which are expected to pave a pathway towards self-powered electronics for locomotor behavior analysis and sports management.

## Data Availability

All data supporting the findings of this study are available within the paper and its Supplementary Information.

## Supplementary Materials

The following supporting information can be downloaded at: https://www.mdpi.com/article/10.3390/s25103232/s1, Figure S1: Photos of IRMOF-1 and IRMOF-3 crystals; Figure S2: FT-IR spectra and XRD patterns of IRMOF-1 and IRMOF-3 crystals; Figure S3: AFM image of CNFs produced by TEMPO oxidation and the cross-sectional profile; Figure S4: Photos of CNF/IRMOF-1 and CNF/IRMOF-3 suspensions; Figure S5: Photograph of CNF/IRMOF-1 composite film prepared by conventional evaporation method and optimized method using ZnCl_2_ as the CNF matrix stabilizer; Figure S6: Surface morphology maps of CNF/IRMOF-1 and CNF/IRMOF-3 surfaces; Figure S7: The output curves of open-circuit voltage and short-circuit current of CNF/IRMOF-3-based TENG under different humidity conditions in natural environment. Video S1: CI-3 0.6 wt.% TENG as an energy harvester to light up LED bulbs real-time human motion monitoring; Video S2: CI-3 0.6 wt.% TENG as an indicator for walking measurement. Video S3: CI-3 0.6 wt.% TENG as an energy harvester to drive small electronic device.

## References

[1] Luo J., Gao W., Wang Z.L. (2021). The Triboelectric Nanogenerator as an Innovative Technology toward Intelligent Sports. Adv. Mater..

[2] Liu S., Tong W., Gao C., Liu Y., Li X., Zhang Y. (2023). Environmentally friendly natural materials for triboelectric nanogenerators: A review. J. Mater. Chem. A.

[3] Li N., Qiao L., He J., Wang S., Yu L., Murto P., Li X., Xu X. (2021). Solar-Driven Interfacial Evaporation and Self-Powered Water Wave Detection Based on an All-Cellulose Monolithic Design. Adv. Funct. Mater..

[4] Wu J., Li X., Xue N., Wang J., Xu G., Chen S., Cui H., Zi Y., Wang Z.L. (2025). Managing the two mode outputs of triboelectric nanogenerators to reach a pulsed peak power density of 31 MW m^−2^. Energy Environ. Sci..

[5] Wang F., Wang S., Liu Y., Hou T., Wu Z., Qian J., Zhao Z., Wang L., Jia C., Ma S. (2024). Improved Electrical Output Performance of Cellulose-Based Triboelectric Nanogenerators Enabled by Negative Triboelectric Materials. Small.

[6] Dong L., Wang M., Wu J., Zhu C., Shi J., Morikawa H. (2022). Stretchable, Adhesive, Self-Healable, and Conductive Hydrogel-Based Deformable Triboelectric Nanogenerator for Energy Harvesting and Human Motion Sensing. ACS Appl. Mater. Interfaces.

[7] Lin C., Zhao H., Huang H., Ma X., Cao S. (2023). PEO/cellulose composite paper based triboelectric nanogenerator and its application in human-health detection. Int. J. Biol. Macromol..

[8] Graham S.A., Patnam H., Manchi P., Paranjape M.V., Kurakula A., Yu J.S. (2022). Biocompatible electrospun fibers-based triboelectric nanogenerators for energy harvesting and healthcare monitoring. Nano Energy.

[9] He X., Zou H., Geng Z., Wang X., Ding W., Hu F., Zi Y., Xu C., Zhang S.L., Yu H. (2018). A Hierarchically Nanostructured Cellulose Fiber-Based Triboelectric Nanogenerator for Self-Powered Healthcare Products. Adv. Funct. Mater..

[10] Ding Z., Tian Z., Ji X., Yang G., Sameer M., Lu Y., Rojas O.J. (2024). Hybrid Cellulose-Based Systems for Triboelectrification in Aerosol Filtration, Ammonia Abatement and Respiration Monitoring. Adv. Funct. Mater..

[11] Zheng T., Li G., Zhang L., Sun W., Pan X., Chen T., Wang Y., Zhou Y., Tian J., Yang Y. (2023). A waterproof, breathable nitrocellulose-based triboelectric nanogenerator for human-machine interaction. Nano Energy.

[12] Zhang S., Xiao Y., Chen H., Zhang Y., Liu H., Qu C., Shao H., Xu Y. (2023). Flexible Triboelectric Tactile Sensor Based on a Robust MXene/Leather Film for Human–Machine Interaction. ACS Appl. Mater. Interfaces.

[13] Fang S., Ji X., Wang H., Jiang H., Gao M., Liu H., Liu Y., Cheng B. (2024). Cellulose-based green triboelectric nanogenerators: Materials, form designs, and applications. J. Mater. Chem. A.

[14] Saito T., Nishiyama Y., Putaux J.-L., Vignon M., Isogai A. (2006). Homogeneous Suspensions of Individualized Microfibrils from TEMPO-Catalyzed Oxidation of Native Cellulose. Biomacromolecules.

[15] Meng X., Cai C., Luo B., Liu T., Shao Y., Wang S., Nie S. (2023). Rational Design of Cellulosic Triboelectric Materials for Self-Powered Wearable Electronics. Nano-Micro Lett..

[16] Liu Y., Fu Q., Mo J., Lu Y., Cai C., Luo B., Nie S. (2021). Chemically tailored molecular surface modification of cellulose nanofibrils for manipulating the charge density of triboelectric nanogenerators. Nano Energy.

[17] Yao C., Yin X., Yu Y., Cai Z., Wang X. (2017). Chemically Functionalized Natural Cellulose Materials for Effective Triboelectric Nanogenerator Development. Adv. Funct. Mater..

[18] Nie S., Fu Q., Lin X., Zhang C., Lu Y., Wang S. (2021). Enhanced performance of a cellulose nanofibrils-based triboelectric nanogenerator by tuning the surface polarizability and hydrophobicity. Chem. Eng. J..

[19] Du G., Wang J., Liu Y., Yuan J., Liu T., Cai C., Luo B., Zhu S., Wei Z., Wang S. (2023). Fabrication of Advanced Cellulosic Triboelectric Materials via Dielectric Modulation. Adv. Sci..

[20] Chen Y., Li D., Xu Y., Ling Z., Nawaz H., Chen S., Xu F. (2022). Surface-microstructured cellulose films toward sensitive pressure sensors and efficient triboelectric nanogenerators. Int. J. Biol. Macromol..

[21] Shao Y., Feng C.-P., Deng B.-W., Yin B., Yang M.-B. (2019). Facile method to enhance output performance of bacterial cellulose nanofiber based triboelectric nanogenerator by controlling micro-nano structure and dielectric constant. Nano Energy.

[22] Kirchon A., Feng L., Drake H.F., Joseph E.A., Zhou H.-C. (2018). From fundamentals to applications: A toolbox for robust and multifunctional MOF materials. Chem. Soc. Rev..

[23] Khandelwal G., Chandrasekhar A., Raj N.P.M.J., Kim S.-J. (2019). Metal–Organic Framework: A Novel Material for Triboelectric Nanogenerator–Based Self-Powered Sensors and Systems. Adv. Energy Mater..

[24] Shaukat R.A., Saqib Q.M., Kim J., Song H., Khan M.U., Chougale M.Y., Bae J., Choi M.J. (2022). Ultra-robust tribo- and piezo-electric nanogenerator based on metal organic frameworks (MOF-5) with high environmental stability. Nano Energy.

[25] Hajra S., Sahu M., Padhan A.M., Lee I.S., Yi D.K., Alagarsamy P., Nanda S.S., Kim H.J. (2021). A Green Metal–Organic Framework-Cyclodextrin MOF: A Novel Multifunctional Material Based Triboelectric Nanogenerator for Highly Efficient Mechanical Energy Harvesting. Adv. Funct. Mater..

[26] Wang T., Zhu Q., Zhu Q., Yang Q., Wang S., Luo L. (2022). A highly stable bimetallic organic framework for enhanced electrical performance of cellulose nanofiber-based triboelectric nanogenerators. Nanoscale Adv..

[27] Mai Z., Liu D. (2019). Synthesis and Applications of Isoreticular Metal–Organic Frameworks IRMOFs-n (n = 1, 3, 6, 8). Cryst. Growth Des..

[28] Eddaoudi M., Kim J., Rosi N., Vodak D., Wachter J., O’Keeffe M., Yaghi O.M. (2002). Systematic Design of Pore Size and Functionality in Isoreticular MOFs and Their Application in Methane Storage. Science.

[29] Bhatta T., Maharjan P., Cho H., Park C., Yoon S.H., Sharma S., Salauddin M., Rahman M.T., Rana S.M.S., Park J.Y. (2021). High-performance triboelectric nanogenerator based on MXene functionalized polyvinylidene fluoride composite nanofibers. Nano Energy.

[30] Wang H., Shi M., Zhu K., Su Z., Cheng X., Song Y., Chen X., Liao Z., Zhang M., Zhang H. (2016). High performance triboelectric nanogenerators with aligned carbon nanotubes. Nanoscale.

[31] Saito T., Isogai A. (2004). TEMPO-Mediated Oxidation of Native Cellulose. The Effect of Oxidation Conditions on Chemical and Crystal Structures of the Water-Insoluble Fractions. Biomacromolecules.

[32] Zhou X., Zhang Y., Yang X., Zhao L., Wang G. (2012). Functionalized IRMOF-3 as efficient heterogeneous catalyst for the synthesis of cyclic carbonates. J. Mol. Catal. A Chem..

[33] Tang W., Zhang C., Han C.B., Wang Z.L. (2014). Enhancing Output Power of Cylindrical Triboelectric Nanogenerators by Segmentation Design and Multilayer Integration. Adv. Funct. Mater..

[34] Zong Y., Wang R., Xu S., Zhang R., Zhang Z. (2023). Flexible Piezoelectric Nanogenerator Based on Cellulose Nanofibril/MXene Composite Aerogels for Low-Frequency Energy Harvesting. ACS Appl. Nano Mater..

[35] Li Y., Luo Y., Deng H., Shi S., Tian S., Wu H., Tang J., Zhang C., Zhang X., Zha J.-W. (2024). Advanced Dielectric Materials for Triboelectric Nanogenerators: Principles, Methods, and Applications. Adv. Mater..

[36] Yu Y., Gao Q., Zhao D., Li X., Wang Z.L., Cheng T. (2022). Influence of mechanical motions on the output characteristics of triboelectric nanogenerators. Mater. Today Phys..

