# Supplementary material for: Cellulose Nanofibril-Based Triboelectric Nanogenerators Enhanced by Isoreticular Metal-Organic Frameworks for Long-Term Motion Monitoring
Source: Sensors (Basel). 2025 May 21;25(10):3232. doi: 10.3390/s25103232 (PMC12115699; doi:10.3390/s25103232)
Supplement: Supplementary file 1 [file sensors-25-03232-s001.zip › sensors-3569176-supplementary.pdf]

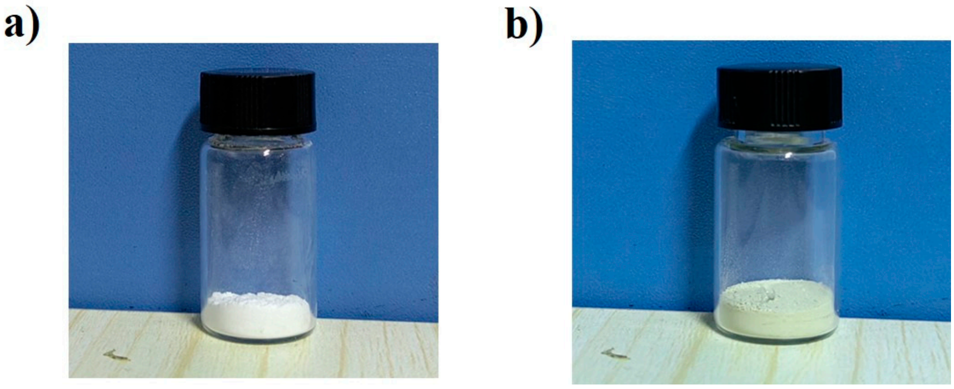

Figure S1. Photos of (a) IRMOF-1 and (b) IRMOF-3 crystals.

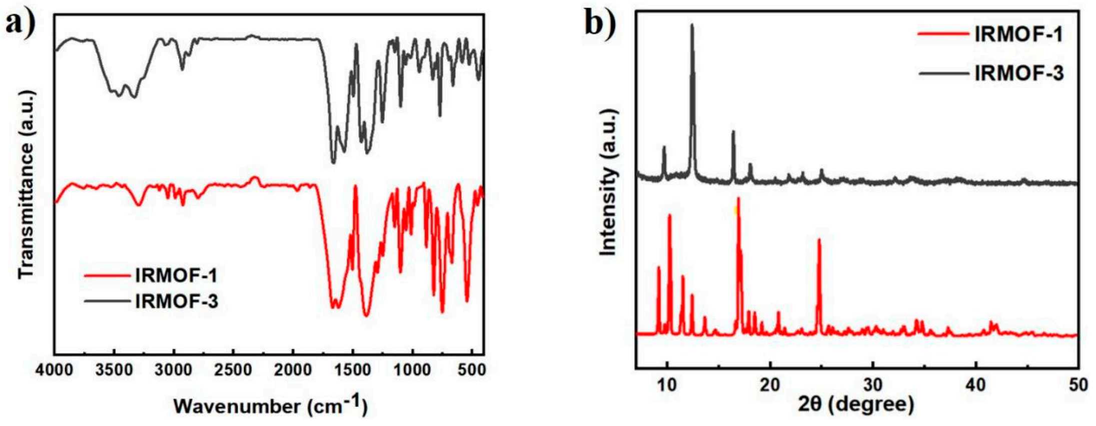

Figure S2. (a) FT-IR spectra and (b) XRD patterns of IRMOF-1 and IRMOF-3 crystals.

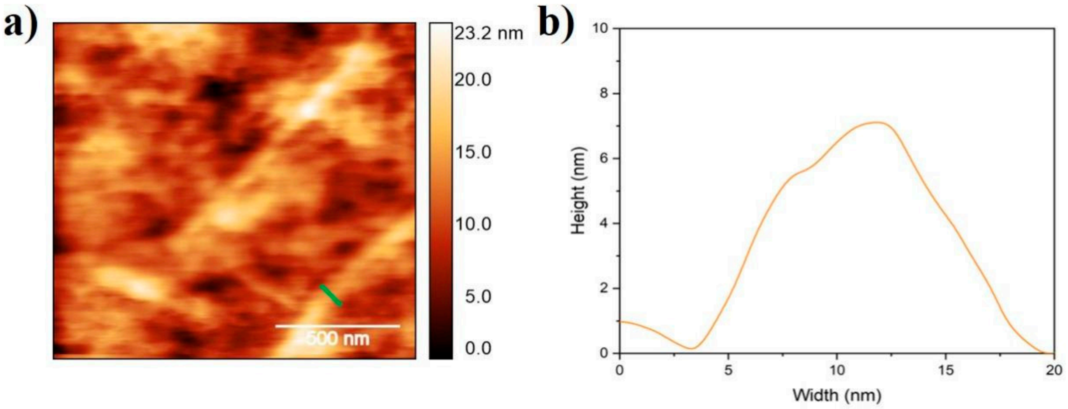

Figure S3. (a) AFM image of CNFs produced by TEMPO oxidation; (b) the cross-sectional profile obtained from the location labeled in green.

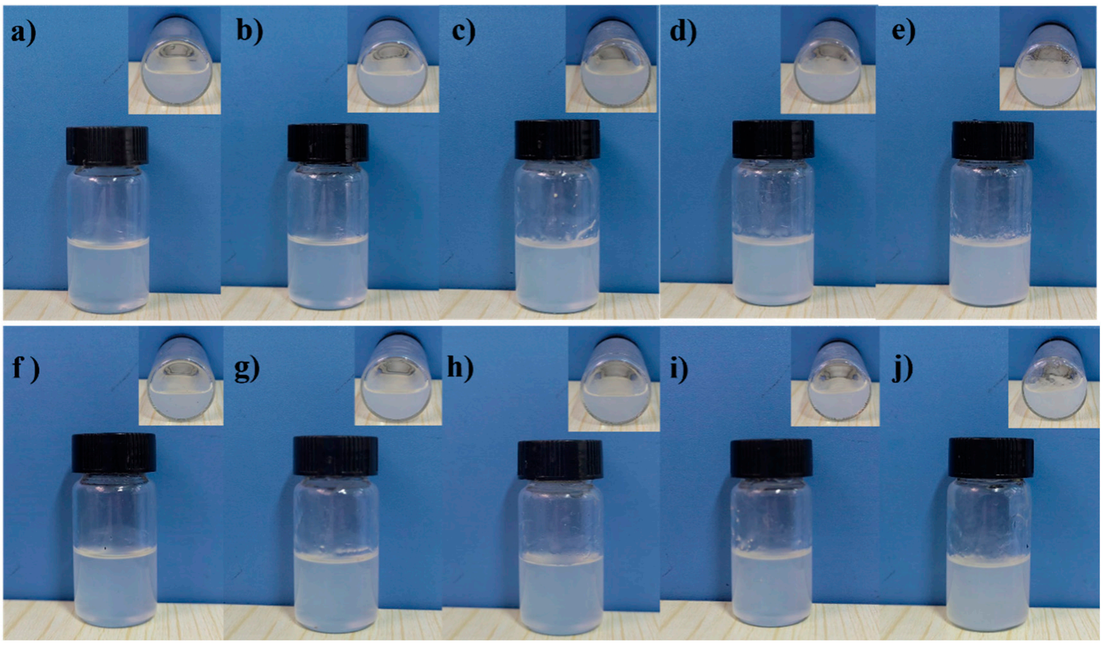

Figure S4. Photos of CNF/IRMOF-1 and CNF/IRMOF-3 suspensions: (a-e) the weight ratio percentages of IRMOF-1 are (a) 0, (b) 0.2, (c) 0.4, (d) 0.6 and (e) 0.8 wt%, respectively; (f-j) the weight ratio percentages of IRMOF-3 are (f) 0, (g) 0.2, (h) 0.4, (i) 0.6 and (j) 0.8 wt%, respectively.

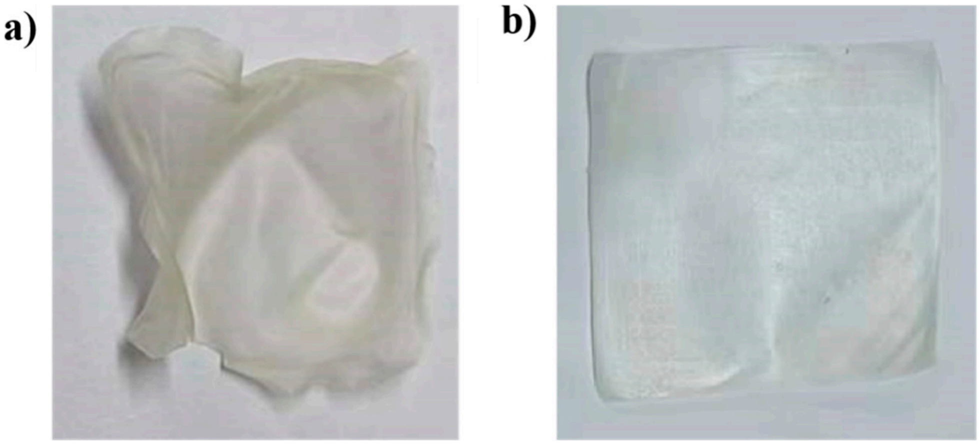

Figure S5. (a) Photograph of CNF/IRMOF-1 composite film prepared by conventional evaporation method; (b) Photo of CNF/IRMOF-1 composite films prepared by optimized method using $ZnCl_2$ as the CNF matrix stabilizer.

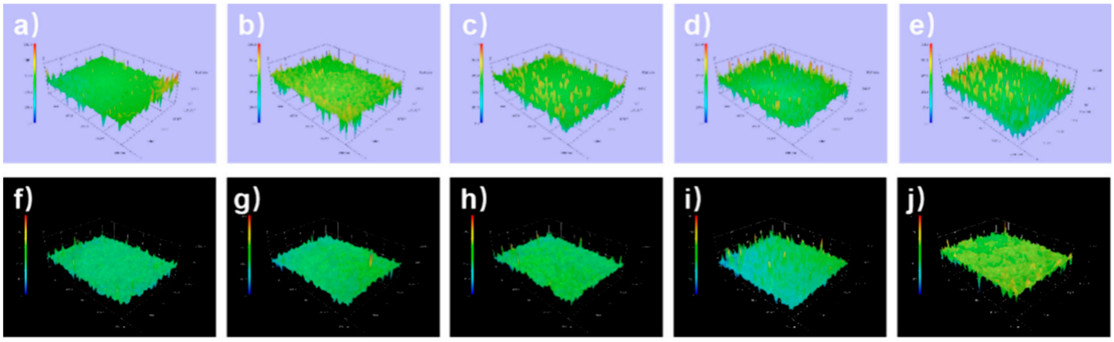

Figure S6. (a-e) Surface morphology maps of CNF/IRMOF-1 surfaces with (a) 0, (b) 0.2, (c) 0.4, (d) 0.6 and (e) 0.8 wt% of IRMOF-1, respectively; (f-j) Surface morphology maps of CNF/IRMOF-3 surfaces with (f) 0, (g) 0.2, (h) 0.4, (i) 0.6 and (j) 0.8 wt% of IRMOF-3, respectively.

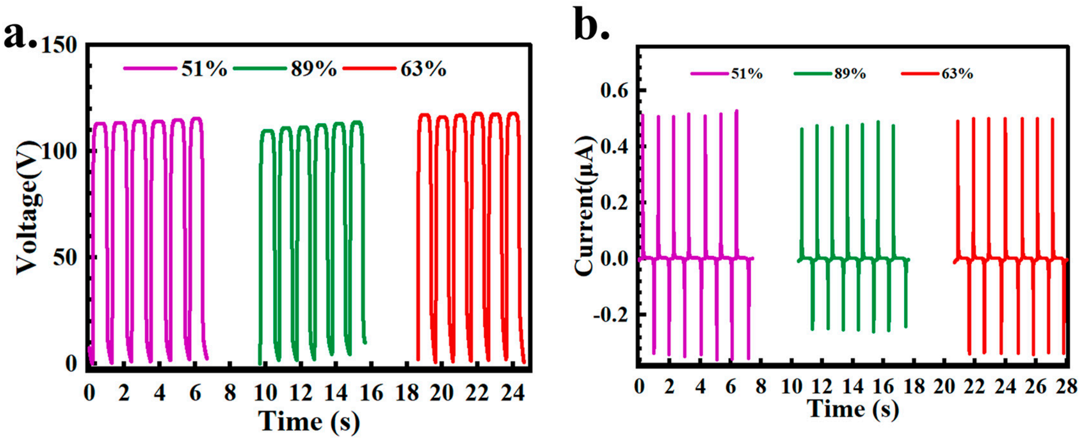

Figure S7. The output curves of (a) open-circuit voltage and (b) short-circuit current of CNF/IRMOF-3-based TENG under different humidity conditions in natural environment.
